# Characteristics of Three-Dimensional Power Doppler in Gestational Trophoblastic Disease

**DOI:** 10.1155/2015/917687

**Published:** 2015-07-16

**Authors:** Wei Wang, Xueye Tian, Ting Zhang, Yanyan Wang, Zhen Han, Ruifang An

**Affiliations:** Obstetrics and Gynecology Department, The First Affiliated Hospital, Xi'an Jiaotong University, Xi'an 710061, China

## Abstract

*Purpose*. In the present study, the three-dimensional power Doppler was used as a quantitative method to evaluate its reliability in detecting and assessing of gestational trophoblastic disease (GTD). *Methods*. 52 GTD patients who received diagnosis and treatment at the first affiliated hospitals of Xi'an Jiaotong University in China between 2011 and 2013 were evaluated using Voluson E8 (GE Medical System). Demographic information, pathological characteristics, clinical history, sonographic images, and related indices (resistance index, vascularization index, and flow and vascularization index) were evaluated. *Result*. Three-dimension power Doppler indicated that there were significant differences in the resistance index, vascularization index, flow index, and vascularization-flow index between the healthy individuals and each subgroup of patients (*P* < 0.01). Further, in combining invasive hydatidiform mole and choriocarcinoma groups, there was a significant difference between hydatidiform mole and the combined malignant group (*P* < 0.01). And the abnormal sonographic and power Doppler findings in GTD were resolved when chemotherapy was done successfully. *Conclusion*. Combined with the clinical features, sonography and three-dimension power Doppler imaging were helpful in diagnosing GTD as a noninvasive method, distinguishing the invasive nature of disease, detecting the recurrence of the disease, and assessing the effectiveness of the chemotherapy.

## 1. Introduction

Gestational trophoblastic disease (GTD) is a rare gynecological disease and contains a series of pregnancy-related disorders, which occurs in the delivery, miscarriage, or termination of pregnancy. It consists of the premalignant tumor of complete and partial hydatidiform mole, which might develop into invasive mole, choriocarcinoma, and the rare placental-site trophoblastic tumor in rare cases [1]. GTD derives from the healthy trophoblastic tissue that can be invaded gradually to become a luxuriant uterine vasculature and can be detected by polymerase chain reaction (PCR) in the maternal circulation [2]. The early clinical manifestations are menolipsis, vaginal bleeding, and the abnormal rising of human chorionic gonadotrophin (hCG) and minority is diagnosed by the first symptoms of cough and headache which are caused by lung and brain metastasis, respectively. The level of *β*-hCG is a biomarker to diagnose, evaluate, and supervise the final outcome of GTD. In China, serum *β*-hCG concentrations are measured every week until the values are within the normal range and recorded monthly. However, related studies show that there are large differences between assays in recognition of isoforms of hCG [3]. Transvaginal ultrasonography has become a powerful method to assess the form, volume, and color flow mapping of the uterine and ovarian layer. The power Doppler has been extensively used in understanding the uterine vasculature. Three-dimensional (3D) ultrasound estimates Doppler signals throughout a whole organ or area, so it may be more reflective of vascularization and blood flow which is dissimilar to conventional two-dimensional ultrasound [4, 5]. Blood flow velocity waveform index refers to any index calculated from a Doppler shift spectrum like pulsatility index (PI), resistance index (RI), and systolic diastolic ratio (S : D ratio). Moreover, through the 3D vascular indices, that is, vascularization index (VI), flow index (FI), and vascularization-flow index (VFI) by using virtual organ computer-aided analysis (VOCAL) view generate a noninvasive tool to describe the indices of vascularity [6, 7]. In this study, the 3D power Doppler (3D PD) was used as a quantitative method in order to understand its reliability in detecting and following GTD.

## 2. Materials and Methods

### 2.1. Clinical Characteristics

We prospectively collected the data of 52 patients diagnosed with GTD from the database at the First Affiliated Hospital of Xi'an Jiaotong University from December 2011 to October 2013. The data of these patients included demographic parameters, histopathologic tumor characteristics, inspection result of sonography and combined *β*-hCG, and complete remission time. The eligibility criteria were (i) patients without a history of prior GTD or other malignancies and (ii) patients who complied with the treatment and follow-up. 30 healthy women (from the health examination center of the First Affiliated Hospital of Xi'an Jiaotong University from December 2011 to October 2013) aged 18 years or over were included in the study on a voluntary basis as control. The blood flow parameters of the control group were collected randomly from 5 views from the uterus and then calculated as mean value.

### 2.2. Sonography Technique

**Figure 1:**
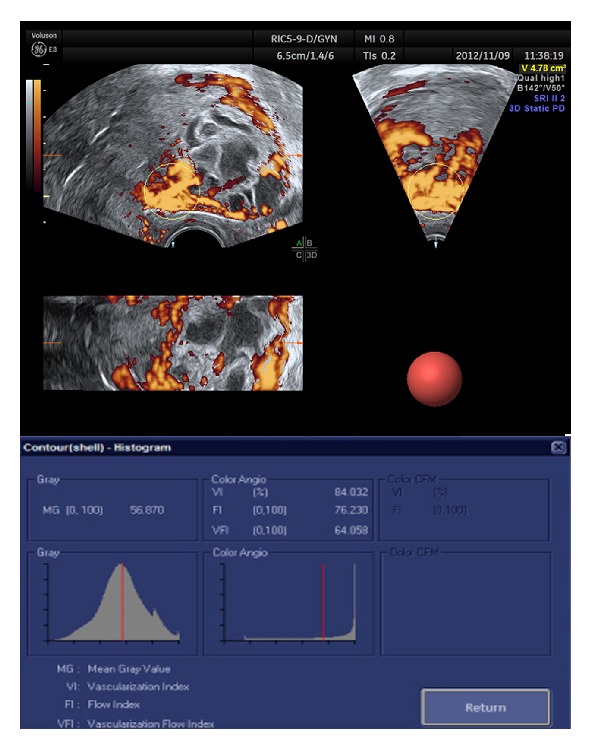
VOCAL automated analysis of a GTD patient.

Sonography was performed using a Voluson E8 (GE Medical Systems) with a transvaginal RIC5-9D 3D curved array transducer. A single observer (HZ) was performed for all the scans. The details to diagnosis and assessing GTD were a complex echogenic uterine mass with numerous cystic spaces and no fetus or amnionic sac for complete mole, a thickened and hydropic placenta with fetal tissue for partial mole and nonspecific focal masses with myometrial epicenter which were sonographically indistinguishable from one another for invasive mole and choriocarcinoma. Primary two-dimensional (2D) ultrasound assessment was routinely performed to determine uterine size, myometrium hemodynamics parameters, and blood sinus resistance index (RI). After preliminary 2D sonography, the region of interest was defined as the area of the myometrium. The following detailed settings were kept identical for all examinations since any modification of the setting would affect the resultant VOCAL indices: Gain was at −0.0, power was “100%,” wall motion filter was at “low 1,” PRF was set at “1.5 KHz,” frequency was “mid,” flow resolution was “high,” and quality was “high.” Five sets of data were collected per case. From the stored data, analysis of acquired volumes about a thickness of 1 cm was performed by a second observer (WW) using 4D View (Version 10.2; GE Healthcare) for VOCAL automated analysis. VI, FI, and VFI of the region of interest were collected for further analysis (Figure 1). VI measures the number of color voxels in the region of myometrium representing the vessels in the tissue and is expressed as a percentage; FI indicates the mean color value in the color voxels and represents the average intensity of flow; and VFI is the mean color value of all the voxels in the sphere, representing both vascularization and flow [8].

### 2.3. Statistical Analysis

Statistical analyses were performed using SPSS 13.0. Consecutive data were presented as mean ± standard deviation (SD). Categorical data were compared by *χ* ^2^ test or Fisher's exact test. The means of the two groups were assessed with rank-sum test or *t*-test. All statistical tests were two-sided, and *P* values < 0.05 were considered to be statistically significant.

## 3. Results

**Figure 2:**
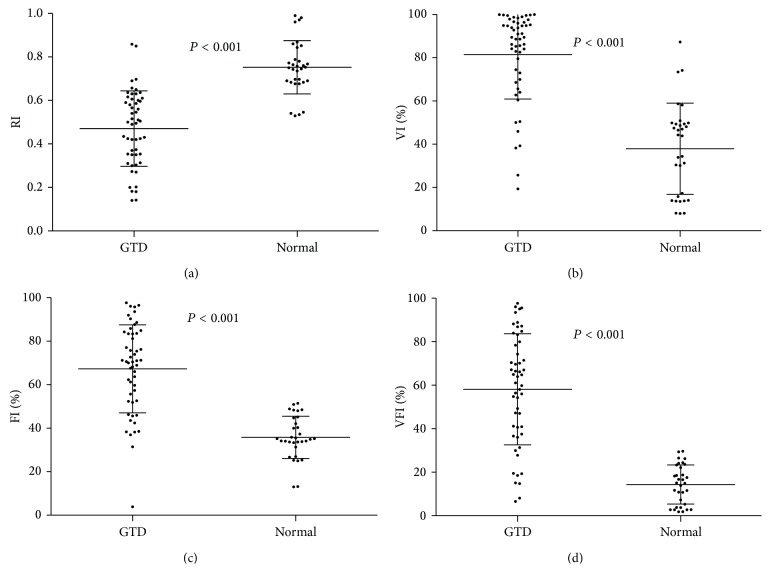
The value of RI, VI, FI, and VFI between normal individuals and GTD patients (*P* < 0.001).

**Figure 3:**
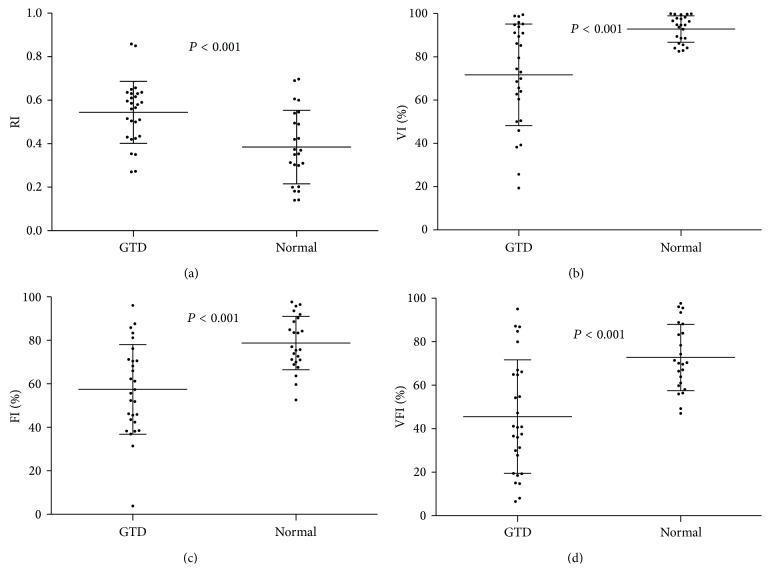
The value of RI, VI, FI, and VFI between hydatidiform mole and combined malignant patients (*P* ≤ 0.001).

The study comprised 52 females with a medium age of 30 (ranged from 21 to 55) years. 28 patients were diagnosed as hydatidiform mole, 20 patients were diagnosed with invasive mole, and 4 patients were diagnosed with choriocarcinoma. All of these tumors were diagnosed by sonography and/or pathological examination. For invasive mole and choriocarcinoma patients, they were followed until they reached complete remission after chemotherapy (EMA-CO or 5-Fluorouracil based chemotherapy). Of the 52 GTD patients history, variation from the mean value was as follows: RI was 0.47 ± 0.17 (ranged from 0.14 to 0.86); VI was 81.46% ± 20.54% (ranged from 19.37% to 99.95%); FI was 67.28% ± 20.21% (ranged from 3.81% to 97.67%); and VFI was 58.12% ±25.53% (ranged 6.55% to 97.62%). The details of each subtypes were shown in Table 1. Meanwhile, the variations from the mean value of normal individuals were as follows: RI was 0.75 ± 0.12 (ranged from 0.53 to 0.99); VI was 37.91% ± 21.09% (ranged from 7.99% to 87.28%); FI was 35.78% ± 9.74% (ranged from 13.00% to 51.41%); and VFI was 14.33% ± 8.97% (ranged from 1.80% to 29.64%). There were significant differences in RI, VI, FI, and VFI between normal individuals and GTD patients (Figure 2). Next, we combined invasive mole with choriocarcinoma as the malignant group and found that RI, VI, FI, and VFI were also the factors which could help distinguishing the hydatidiform mole and malignant GTD (Figure 3). They can provide some corroborative bodies of evidence in distinguishing the hydatidiform mole and malignant GTD. Therefore, it can be concluded that low RI and high VI, FI, and VFI were found commonly in malignant GTD.

**Figure 4:**
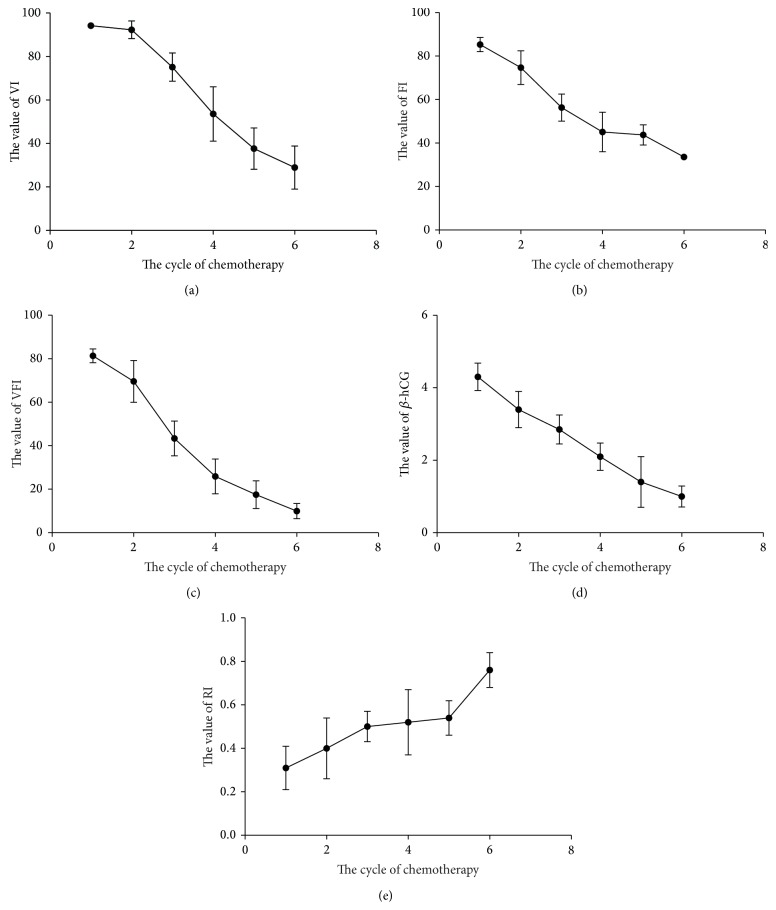
The tendency of *β*-hCG, RI, VI, FI, and VFI during the chemotherapy. (a) The value of VI (%) was decreasing coincidence with *β*-hCG. (b) The value of FI (%) was decreasing coincidence with *β*-hCG. (c) The value of VFI (%) was decreasing coincidence with *β*-hCG. (d) The value of *β*-hCG (Log_10_ *β*-hCG) was declining during the chemotherapy. (e) The value of RI was recovering with fluctuation.

Furthermore, 19 of the 24 patients in the malignant group received chemotherapy and complied with the follow-up. Among the 19 patients with malignant GTD, 16 patients had invasive mole and 3 patients had choriocarcinoma. The values of VI, FI, and VFI were decreasing coincidence with *β*-hCG after chemotherapy in the 19 patients. And the value of RI was recovered while *β*-hCG was going down (Figure 4). Next, we identified the upper limit of 95% confidence interval of VI, FI, and VFI in normal individuals as the normal border (95%CI of VI, 30.31% to 40.51%; 95%CI of FI, 32.27% to 39.29%; 95%CI of VFI, 11.09% to 17.56%). We found that the value of VI, FI, and VFI of 18 patients reached the normal level when their *β*-hCG reached the normal level (less than 40.51% for VI, 39.29% for FI, and 17.56% for VFI).

## 4. Discussion

Despite *β*-hCG, ultrasound, as a convenient and repeatable noninvasive screening method, is widely used in the early diagnosis and assessing the outcome of GTD. Typical characteristics of color Doppler flow imaging in myometrium are rendered as a focal point honeycomb echo area which is rich in blood flow, irregular shape, and colorful mosaic sphere with bright colors. Compared with traditional two-dimensional images, the focal color flow of honeycomb change occurs early and fades later. It can also clearly identify the scope and depth of the lesion which is conducive to clinical follow-up and early detection of GTD [9]. In a GTD patient, invasion of myometrial arteries by the trophoblastic tissue occurs, and this process is accentuated by the abnormal trophoblastic proliferation. In the earlier studies, the sonography analysis shows evidence of high-velocity, low-impedance flow in the GTD patients [9]. So combined with the elevated *β*-hCG level, RI can be a marker to diagnosis GTD, evaluate the therapeutic effects, and provide some imaging confirmation of GTD.

In our study, we evaluated VI, FI, and VFI using VOCAL automated analysis. We found that VI, FI, and VFI not only can be a screening tool in detecting GTD but also can be a marker in assessing the outcome of GTD patients. The results of our study indicate that there are significant differences in VI, FI, and VFI between normal individuals and GTD patients. And these factors can also help distinguishing the hydatidiform mole and malignant GTD. This shows that 3D PD is more sensitive than traditional color Doppler in the assessment of GTD and possible to objectively quantify the blood resistance, vascularization, and flow. This is partly due to the situation of blood flow and VOCAL program which applies a rotational method. Previous studies have shown that VI, FI, and VFI could be a helpful tool to detect the malignancies, such as endometrial neoplasms and ovarian tumor [10, 11].

Furthermore, we found that the VI, FI, and VFI were decreasing coincidence with *β*-hCG after chemotherapy in the 19 patients with malignant GTD. Our study is the first to identify the normal range of VI, FI, and VFI of normal individuals. Based on our data, 18 patients reached the normal border when their *β*-hCG drops to normal level. The patient, who did not reach the normal level after chemotherapy, revealed that VI was 45.21%, FI was 47.53, and VFI was 23.46%. And this patient received more chemotherapy (12 times) and might need additional course of chemotherapy. The vascularization and the flow manifest the outcome of GTD. This might indicate that these three factors can be the factor to assess the therapeutic effect and provide three-dimensional image data to clinicians.

As a rare disease, a multicenter study may be needed to make a more accurate threshold value of blood flow indexes. At the same time, it is a great problem to avoid cavitation effect during the 3D PD examination for the patients with early pregnancy. Further, power Doppler quantification is currently considered a limited tool to assess vascularization, mainly because of the high dependence of gain and other machine settings, attenuation, and reproducibility. So, skilled sonographers are needed in order to shorten the 3D PD examination and make a more accurate and reproductive result. Moreover, fractional moving blood volume (FMBV), as a quantitative methodology that compensates for common estimation errors, may need to reduce the dependency of machine settings and attenuation.

We conclude that 3D PD can be an accessory tool to support the diagnosis of GTD and assess the outcome of chemotherapy in GTD patients. The VI, FI, and VFI can be the parameters in GTD screening.

## Figures and Tables

**Table 1:** The characteristics of subgroups of GTD.

	Hydatidiform mole	Invasive mole	Choriocarcinoma	*P* value
	*n* = 28; mean ± SD	*n* = 20; mean ± SD	*n* = 4; mean ± SD	
Age (year)	30.79 ± 7.82	29.55 ± 6.56	29.25 ± 7.59	0.875
RI	0.54 ± 0.14	0.41 ± 0.17	0.25 ± 0.06	0.001
VI	71.69% ± 23.47%	92.36% ± 6.58%	95.38% ± 1.38%	0.002
FI	57.44% ± 20.64%	77.44% ± 12.86%	85.30% ± 6.47%	<0.001
VFI	45.58% ± 26.09%	71.04% ± 15.98%	81.36% ± 6.36	<0.001
